# Geographic proximity to Down syndrome specialty clinics for individuals born with Down syndrome, 1999–2020 in Texas

**DOI:** 10.1371/journal.pone.0361583

**Published:** 2026-10-28

**Authors:** Henal A. Gandhi, Dayana Betancourt, Charles Shumate, Rachel Allred, Ashley Dixon, Madeline Kilburn, Claire Singletary, Daniel Riconda, A. J. Agopian

**Affiliations:** 1 Birth Defects Epidemiology and Surveillance Branch, Texas Department of State Health Services, Austin, Texas, United States of America; 2 Department of Pediatrics and Department of Obstetrics, Gynecology, and Reproductive Sciences, McGovern Medical School at UTHealth Houston, Genetic Counseling Program, The University of Texas MD Anderson Cancer Center UTHealth Houston Graduate School of Biomedical Sciences, University of Texas Health Science Center at Houston, Houston, Texas, United States of America; 3 Department of Molecular and Human Genetics, Baylor College of Medicine, Houston, Texas, United States of America; 4 Department of Epidemiology, University of Texas Health Science Center at Houston (UTHealth) School of Public Health, Houston, Texas, United States of America; The University of Alabama in Huntsville, UNITED STATES OF AMERICA

## Abstract

**Background:**

While effective national clinic networks exist for a select few conditions, the availability and use of multidisciplinary specialty care for most genetic or congenital conditions, including Down syndrome (DS), remain uneven. This uneven distribution creates significant geographic and social gradients in healthcare access. To understand these differences within a geographically expansive and socio-demographically diverse setting, this study quantified travel burden to pediatric DS specialty clinics in Texas, identifying differences based on key maternal socio-demographic and geographic factors.

**Methods:**

A cross-sectional analysis of 8,201 individuals with DS presumed living and 477 deceased individuals with DS born between 1999–2020 from the Texas Birth Defects Registry was performed. One-way driving time and distance along the road networks from the maternal residence to the nearest seven pediatric DS specialty clinics were calculated using ArcGIS Pro. Travel burden was analyzed by maternal race/ethnicity, education, age and residence at delivery. Travel burden measures were categorized as low, moderate to high, and very high.

**Results:**

Among 8,678 live‑born individuals with DS, 45% resided over 90 miles from the closest in-state DS specialty clinic, and 41% faced one‑way driving times exceeding two hours. Those born to mothers in border counties experienced the greatest travel burden, with median one-way driving times exceeding 7 hours. Rural residents had a four times greater travel burden than urban residents. Individuals born to Hispanic mothers traveled twice as far as those born to non-Hispanic mothers. Residents of high‑poverty tracts had a median one-way distance of approximately 150 miles, triple the 35 miles seen in low‑poverty tracts.

**Conclusion:**

Our findings suggest that substantial travel burden to DS specialty clinics exists in Texas, affecting individuals with DS born to Hispanic and younger mothers, as well as those in rural, border, and concentrated-poverty regions. Further research is needed to explore life course residential history and clinic utilization to address these differences.

## Introduction

Multidisciplinary specialty clinics play a crucial role in delivering high-quality care for children with congenital conditions, including Down syndrome (DS), orofacial clefts, Critical Congenital Heart Defect (CCHD) and spina bifida among others [1,2]. Although centralized condition-specific care models have been successfully established for certain genetic or congenital conditions like cystic fibrosis, the broader landscape for most congenital conditions lacks such infrastructure [3,4]. Consequently, the availability and utilization of specialized care remain highly variable, leading to stark geographic and social differences in access at both individual and population levels [5–7]

In the United States, DS is the most common chromosomal aneuploidy with a prevalence of approximately 15 per 10,000 live births [8,9]. Texas had 11,694 babies born with DS between 1999 and 2020, corresponding to an annual prevalence of 13.85 per 10,000 live births [10]. Advances in medical care have improved life expectancy for individuals with DS [11,12]. However, individuals with DS remain at increased risk for comorbidities, including congenital heart defects, thyroid dysfunction, sensory impairments, epilepsy, sleep disorders, leukemia, Alzheimer’s disease, and metabolic conditions [13–21]. These conditions benefit from multidisciplinary care that extends beyond what primary care providers can deliver.

Families of children with DS often spend years navigating services that operate in isolation from one another, with little coordination or support to guide them [16,22]. Specialized DS clinics have emerged as one model for coordinating the multidisciplinary care needs of individuals with DS. In DS specialty care clinics, multidisciplinary care teams collaborate with individual patients and families on a comprehensive care plan [5]. The team of specialists seeing a patient with DS may include a physician, nutritionist, speech therapist and a social worker along with subspecialists in areas such as cardiology, genetics, and neurology, and referrals to additional specialties are arranged as needs arise [5,23]. Such centers are pivotal not only for ongoing health maintenance but also for the early detection and treatment of the secondary conditions that are prevalent in this population. Furthermore, prior work documents substantial gaps in preventive care among patients who do not receive care from a DS specialty clinic. Fewer than 10% of children and less than half of adolescents with Down syndrome in primary care clinics were up to date on preventive measures such as vaccines, well-visits, and all recommended screenings prior to establishing care at a DS specialty clinic [2,24]. Such models are not unique to the United States. Multidisciplinary DS-focused clinics have been established in a number of countries, including Canada, the Netherlands, Taiwan, Israel, UAE, and South Africa, and several countries have issued national clinical guidelines for DS care [25–33]. These clinics vary in organization and scope but generally seek to coordinate condition-specific specialty referrals and longitudinal care for individuals with DS, and expanding access to coordinated care has been identified as global priority for this population [22]. Domestically, the American Academy of Pediatrics (AAP) provides clinical guidance for age-based screening and management of children and adolescents with DS [34], a framework that DS specialty centers are equipped to implement effectively [2,35,36]. Despite the availability of these guidelines, awareness remains limited both among primary care providers as well as families, with only about half of parents informed about these specialized recommendations [24,35,37,38]. Beyond limited awareness, whether families can physically reach a clinic strongly shapes use of services.

A primary determinant of access is the travel burden imposed on families, a factor governed by the well-documented principle of “distance decay” [39,40]. This concept describes the inverse relationship where the likelihood of utilizing a healthcare service decreases as the driving distance or time required to reach it increases, often resulting in worse health outcomes [39,41]. This effect can begin at surprisingly short thresholds, with some studies noting a decline in utilization with driving times of just 30 minutes [40]. Driving time is often considered a more appropriate metric than linear distance because it better reflects real-world barriers such as road networks and congestion especially relevant in large states like Texas where specialty care is concentrated in urban centers. National data shows that an estimated 20% of individuals with DS face significant geographic barriers to care, and over a third of those in the southern US report driving times exceeding two hours to reach a clinic [5]. These geographic barriers occur within a larger pediatric specialty-care landscape marked by uneven workforce distribution and variable network adequacy in the United States. Pediatric subspecialists remain geographically maldistributed, and travel burden is a recognized component of access, particularly in rural areas [42–45]. Recent national data indicate that 53% of primary care pediatricians identify travel time as a moderate or significant barrier to subspecialty care, increasing to 86% among rural pediatricians [44]. Pediatric specialty networks may also be narrower than adult networks, reflecting both limited specialist supply and network exclusion [46], while network adequacy standards do not necessarily translate into improved specialty-care access for children with special health care needs [47]. Academic medical centers may serve both local patients and children, particularly those with greater medical complexity, who have been shown to travel longer distances for care [48].

In this study, we evaluated the geographic proximity of pediatric DS specialty clinics for individuals born with DS in Texas between 1999 and 2020. Texas faces unique challenges due to the limited number of clinics distributed throughout its extensive territory, and most children in Texas live more than an hour from the nearest facility providing pediatric emergency or specialty care [45]. As the second largest state in the United States, Texas exhibits significant socio-demographic variation. The state contains several of the country’s largest metropolitan centers, alongside extensive rural areas, and a 1,254-mile international border with Mexico, as well as substantial racial/ethnic, linguistic, and socioeconomic diversity. For example, more than one-third of Texans speak a language other than English at home, compared with roughly one fifth nationally, and the state includes substantial Hispanic, foreign-born and low-income populations, with a higher proportion of residents living in poverty than the United States overall [49,50]. Understanding the geographic distribution and proximity to clinics across such a varied landscape is crucial for ensuring that all individuals with DS receive the specialized care they need. Accordingly, we described one-way driving times and distances to the nearest pediatric DS specialty clinic in Texas and examined how maternal socio-demographic and geographic factors were associated with geographic proximity. Using DS as a sentinel example of multidisciplinary specialized care, our findings may guide policies and planning aimed at enhancing access to coordinated clinics for a wider array of congenital and developmental conditions at the population level.

## Methods

### Study population and data sources

We conducted a population-based, cross-sectional analysis using the data from the Texas Birth Defects Registry (TBDR). The TBDR is a population-based, active birth defects surveillance registry employing standardized case-finding protocols and medical record abstraction to ascertain cases among all pregnancy outcomes, including live-born infants with birth defects diagnosed between the prenatal period to age one. Data are routinely linked to state vital records for mortality data and to supplement sociodemographic data. Methods of the TBDR have been described elsewhere [51].

The study population included all live-born infants with a confirmed diagnosis of DS made through cytogenetic analysis (including amniocentesis or chorionic villus sampling) with or without known karyotype and identified using six-digit Centers for Disease Control and Prevention (CDC) modified British Paediatric Association (BPA) codes (758.000 to 758.090) delivered to Texas-resident mothers between 1999 and 2020. Individuals with neonatal deaths (within 28 days of birth) were excluded, given their unlikely need for outpatient specialty care.

Two cohorts were analyzed: presumed living individuals aged 18 years and younger at the time of analysis with no documented death and individuals with a documented post-neonatal death (28 days or later). The primary analyses were conducted among those with no documented death. For this cohort, the analysis was restricted to individuals aged 18 years or younger because updated residential address information after birth was unavailable and therefore maternal residence at birth was used as a proxy for current address. This proxy was considered more appropriate for children and adolescents than for adults. To address the possibility that distance to nearest specialty clinic could have contributed toward increased risk of a post-neonatal death, we also conducted secondary analyses among those with a documented indication of death after the neonatal period. The vast majority of individuals in this group died before adulthood and only 2% (9/477) survived to 20 years of age.

### Ethics statement and data access

The Texas legal statute authorizes the TBDR program staff to access data from the registry for the purpose of monitoring trends, supporting research, and connecting families to helpful services. This study was conducted as a non-contact analysis of existing registry data and linked vital records that are routinely collected under state public health authority. No direct contact with individuals or families occurred, and no new data were collected specifically for research purposes. Throughout the study only the program staff who were part of this project had access to the data. In accordance with the institutional and state policies, this analysis of existing surveillance data by the program staff did not require Institutional Review Board (IRB) review and approval because it did not constitute human subjects’ research.

Data were accessed for this study between October 1st, 2024, and October 31st, 2024. Direct identifiers such as names and full residential street addresses were retained only within the secure registry environment and were not included in the final analytic dataset. Following geocoding, all data were de-identified for statistical analysis.

### DS specialty clinic identification

DS specialty clinics in Texas were identified based on the limited literature available on DS specialty clinics in the United States [2,23,35]. Briefly, we searched national advocacy resources, hospital websites, state health agency materials, and used targeted keyword queries to identify DS specialty clinics (Fig 1). For the purpose of this study, DS specialty clinics were defined as either (1) Clinics that primarily function as dedicated primary care providers exclusively for individuals with DS, offering comprehensive care tailored to their specific needs or (2) Clinics that operate as tertiary referral centers, typically situated within larger divisions such as genetics, developmental-behavioral pediatrics, neurodevelopmental or neurology, endocrinology, cardiology, etc., where they are well-equipped to provide specialized care for DS patients and facilitate relatively easy referrals to other necessary specialties within the network. Of these, clinics were only included if they provided multidisciplinary or dedicated DS-focused care with a physical location in Texas and served pediatric patients. Each clinic’s eligibility was confirmed by three clinical reviewers and matched to the period when each patient could have accessed care.

**Fig 1:**
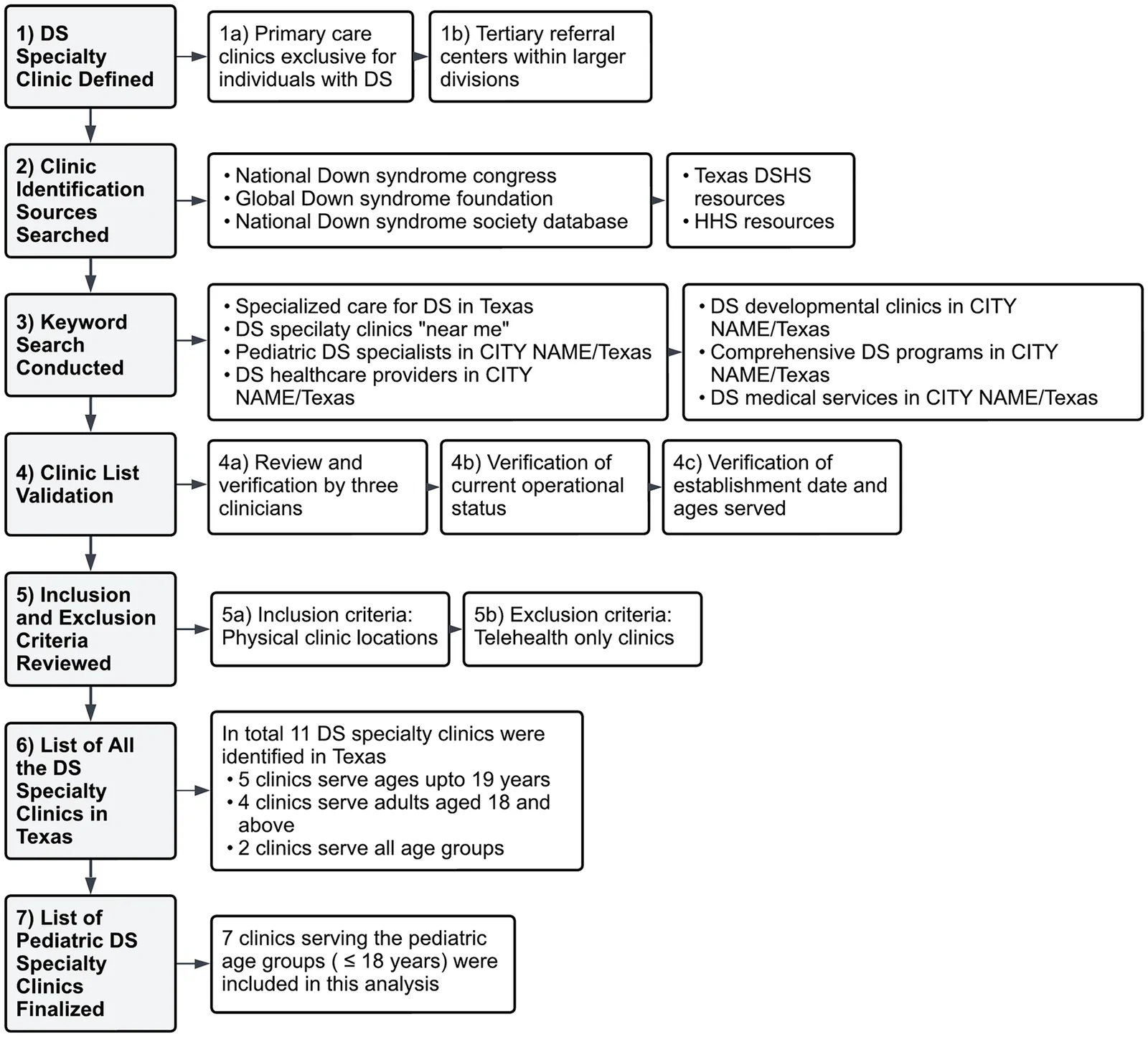
Down syndrome (DS) specialty clinic selection process.

### Geocoding and travel burden estimation

Maternal residential street addresses at delivery and pediatric DS specialty clinic street addresses were geocoded using Esri ArcGIS Pro 3.1. Maternal street addresses were verified from both vital records and medical chart abstraction, defaulting to vital records when discrepancies arose. Geocoding followed an accuracy threshold of 85% or higher with <100 meters positional error [44,52,53]. Addresses below this threshold were manually reviewed. Each maternal street address was spatially linked to the appropriate U.S. Census tract. Road network analysis was performed using ArcGIS Pro’s Network Analyst Extension’s Closest Facility Solver tool [54] to estimate one-way driving distance (miles) and time (minutes) along real-world routes, accounting for legal turns, one-way restrictions, and posted speeds.

### Measures

The primary outcomes were one-way driving time and driving distance to the nearest pediatric DS specialty clinic in Texas. These were categorized into descriptive levels of travel burden by one-way driving distances (d) as Low/local: ≤ 30 miles, Moderate to High: > 30 miles d ≤ 90 miles, and Very High: > 90 miles. The one-way driving time (t) was categorized as Low/local: t ≤ 30 mins, Moderate to High: > 30 mins t ≤ 2hrs, and Very High: > 2hrs consistent with prior literature that has summarized specialty care access using categorized travel time intervals (5,6). Independent variables were derived from registry-linked maternal and census-tract data. These included maternal age, categorized as 35 years and younger or more than 35 years consistent with conventional maternal age groupings used in perinatal and birth defects research; maternal race/ethnicity grouped into White Non-Hispanic (NH), Black NH, Hispanic, and the remaining race/ethnicity groups were grouped together in the NH Additional category; and mother’s education at delivery, classified as less than high school, high school diploma or equivalent, or greater than high school. Geographic factors included urban/rural residence, determined by Rural Urban Continuum Codes [55] and border/non-border county status, using both a broad 32-county and a narrow 14-county definition for the Texas-Mexico border region. Concentrated poverty was defined as maternal residence in a census tract in which more than 20% of the population was living below the federal poverty level, based on the appropriate decennial census and American Community Survey poverty estimates [56]. Father’s demographics were not assessed due to a high level of missingness. Child-specific variables included the child’s age group (4–8, 9–13, and 14–18 years for presumed living children, and 29 days-5 years, 6–12, and 13–20 years for age at death) and the presence of twelve CCHDs, identified via a definitive diagnosis based on specific BPA codes.

### Statistical analysis

Travel burden by cohort and maternal/child characteristics was summarized, reporting medians and interquartile ranges (IQR) given skewed distributions. Associations between each variable and travel burden measures (one-way driving time and distance) were assessed using Wilcoxon rank-sum tests (for binary predictors) and Kruskal Wallis tests (for categorical predictors with more than two levels) and these results are presented in Tables 1 and 2. Categorized travel burden measures were presented descriptively in Tables 3–6 and these did not include a statistical test. Analyses were conducted in SAS 9.4 (Version 9.4, SAS Institute, Cary, NC). Statistical significance was defined as p < 0.05.

**Table 1:** Median one-way driving distances and times to Down syndrome (DS) specialty clinics for living individuals with DS^†^ by select characteristics, Texas, 1999-2020.

Characteristics	N = 8,201 n (%) ^	Median driving distance to the closest pediatric DS specialty clinic (in miles) Median (IQR)	P value	Median driving time to the closest pediatric DS specialty clinic (in minutes) Median (IQR)	P value
**Individual with DS Age at Analysis** ^**#**^			0.21		0.13
4-8 years	2839 (34.6%)	53.4 (17.1-204.4)		68.6 (31.7-196.0)	
9-13 years	2709 (33.0%)	47.3 (16.5-200.7)		62.4 (30.2-192.1)	
14-18 years	2653 (32.4%)	40.0 (15.7-201.3)		56.5 (29.5-193.1)	
**Co-occurring Critical Congenital Heart Defect (CCHD)**			0.49		0.33
Yes	352 (4.3%)	72.4 (15.6-204.6)		81.4 (31.6-196.9)	
No	7849 (95.7%)	46.4 (16.5-202.1)		62.0 (30.3-193.3)	
**Maternal Age at Analysis** ^**#**^			<.0001^*^		<.0001^*^
≤35	1472 (17.9%)	103.8 (17.5-216.4)		110.1 (32.1-208.8)	
>35	6729 (82.1%)	38.8 (16.2-199.4)		55.7 (30.1-191.5)	
**Maternal Race/Ethnicity**			<.0001^*^		<.0001^*^
White non-Hispanic	2405 (29.6%)	52.4 (22.6-161.2)		67.3 (37.7-158.9)	
Black non-Hispanic	738 (9.1%)	19.6 (13.2-85.5)		34.6 (25.5-91.4)	
Hispanic	4672 (57.5%)	102.2 (15.5-319.1)		109.6 (29.5-278.3)	
Additional non-Hispanic groups	384 (4.7%)	23.6 (15.8-111.8)		39.9 (29.0-114.7)	
**Maternal Education at Delivery**			<.0001^*^		<.0005^*^
Greater than high school	3986 (48.8%)	42.7 (19.3-195.6)		59.5 (34.2-189.5)	
High school	1968 (24.1%)	65.9 (15.5-210.5)		78.3 (28.8-201.0)	
Less than high school	2208 (27.1%)	42.2 (13.9-203.1)		56.7 (27.4-193.8)	
**Maternal Residence at Delivery: Concentrated Poverty of >20%** ^**§**^			<.0001^*^		<.0001^*^
Yes	2003 (24.4%)	143.9 (13.9-440.9)		138.1 (27.7-395.7)	
No	6198 (75.6%)	37.2 (17.6-176.1)		54.8 (31.7-179.4)	
**Maternal Residence at Delivery: Texas-Mexico Border/Non-Border**					
***14 Texas Counties***			<.0001^*^		<.0001^*^
Border	1084 (13.2%)	460.7 (447.1-587.3)		412.5 (400.6-489.0)	
Non-border	7117 (86.8%)	30.2 (15.2-158.8)		47.4 (28.7-158.9)	
***32 Texas Counties***			<.0001^*^		<.0001^*^
Border	1130 (13.8%)	353.7 (342.4-585.4)		412.1 (396.9-487.5)	
Non-border	7070 (86.2%)	29.7 (15.1-157.5)		47.2 (28.6-155.3)	
**Maternal Residence at Delivery: Urban/Rural**			<.0001^*^		<.0001^*^
Urban	7469 (91.1%)	32.9 (15.5-199.0)		50.4 (29.2-190.1)	
Rural	731 (8.9%)	143.9 (88.8-330.3)		147.7 (97.0-293.4)	

† The data reflects individuals who, based on available information, are presumed to be living. This assumption is made with the understanding that the registry may not capture all changes in status over time.

# Analysis was completed in October 2024.

^ Percentages (%) may not total 100% due to rounding or missing data.

*Statistically significant (<0.05). *p* values were calculated using the Wilcoxon rank-sum test for two-level variables and the Kruskal Wallis test for variables with three levels.

§ By census tract, 5-year estimates, American Community Survey 2023.

**Table 2:** Median one-way driving distances and times to Down syndrome (DS) specialty clinics for individuals with DS with post-neonatal death^‡^ by select characteristics, Texas, 1999-2020.

Characteristics	N = 477 n (%) ^	Median driving distance to the closest pediatric DS specialty clinic (in miles) Median (IQR)	P value	Median driving time to the closest pediatric DS specialty clinic (in minutes) Median (IQR)	P value
**Individual with DS Age at Death**			0.75		0.90
29 days-5 years	411 (86.2%)	109.2 (17.9-243.6)		111.1 (32.0-230.8)	
6 years-12 years	37 (7.8%)	82.3 (20.6-223.0)		91.8 (32.3-208.6)	
13 years-20 years	29 (6.1%)	177.1 (20.25-260.5)		163.0 (29.3-229.0)	
**Co-occurring Critical Congenital Heart Defect (CCHD)**			0.48		0.49
Yes	8 (1.7%)	40.2 (20.5-161.9)		55.4 (33.7-161.8)	
No	469 (98.3)	114.4 (28.2-226.8)		116.2 (32.0-230.8)	
**Maternal Age at Death of Child with DS**			0.42		0.57
≤35	273 (57.2%)	120.4 (17.0-312.4)		118.7 (30.6-267.0)	
>35	204 (42.8%)	93.1 (20.3-211.0)		92.4 (33.4-203.6)	
**Maternal Race/Ethnicity**			<.0001^*^		<.0001*
White non-Hispanic	137 (28.7%)	75.0 (24.9-168.4)		79.3 (41.4-175.7)	
Black non-Hispanic	56 (11.7%)	20.3 (12.3-102.0)		32.9 (25.0-106.4)	
Hispanic	266 (55.8%)	198.3 (20.4-401.7)		188.7 (36.0-369.4)	
Additional non-Hispanic groups	18 (3.8%)	24.5 (9.9-46.6)		39.8 (21.7-56.4)	
**Maternal Education at Delivery**			0.43		0.48
Greater than high school	165 (34.6%)	121.2 (14.9-311.8)		120.8 (30.8-267.0)	
High school	133 (27.9%)	134.5 (18.9-301.4)		128.4 (31.7-262.6)	
Less than high school	177 (37.1%)	64.3 (20.6-210.0)		76.0 (36.0-199.8)	
**Maternal Residence at Delivery: Concentrated Poverty of >20%** ^**§**^			0.0030*		0.0019*
Yes	194 (40.7%)	160.5 (15.9-420.2)		165.1 (31.8-379.8)	
No	282 (59.1%)	64.5 (18.9-202.0)		75.2 (32.1-195.9)	
**Maternal Residence at Delivery: Texas-Mexico Border/Non-Border**					
***14 Texas Counties***			<.0001*		<.0001^*^
Border	77 (16.1%)	460.0 (451.8-469.0)		410.9 (401.9-432.2)	
Non-border	400 (83.9%)	47.3 (15.1-176.8)		60.4 (29.5-183.6)	
***32 Texas Counties***			<.0001^*^		<.0001^*^
Border	79 (16.6%)	452.3 (440.1-462.7)		400.1 (386.2-410.2)	
Non-border	398 (83.4%)	46.9 (15.1-176.2)		60.1 (29.5-182.6)	
**Maternal Residence at Delivery: Urban/Rural**			<.0001^*^		<.0001^*^
Urban	417 (87.4%)	62.9 (15.8-210.9)		73.7 (29.7-199.3)	
Rural	60 (12.6%)	244.8 (114.3-365.8)		240.4 (117.8-320.8)	

‡ The data reflects individuals who, based on available information, are deceased. This assumption is made with the understanding that the registry may not capture all changes in status over time.

^ Percentages (%) may not total 100% due to rounding or missing data.

*Statistically significant (<0.05). *p* values were calculated using the Wilcoxon rank-sum test for two-level variables and the Kruskal Wallis test for variables with three levels.

§ By census tract, 5-year estimates corresponding to the year of death.

**Table 3:** Travel burden categories by median one-way driving time for living individuals with DS^†^ by select characteristics, Texas, 1999-2020.

Characteristics	Travel Burden Categories by One-Way Driving Time (N, %) ^		
	Low (≤ 30 minutes)	Moderate to High (> 30 minutes and ≤ 2 hours)	Very High (>2 hours)
**Individual with DS Age at Analysis** ^**#**^			
4-8 years	634 (22.2%)	1014 (35.7%)	1191 (42.0%)
9-13 years	668 (24.7%)	938 (34.6%)	1103 (40.7%)
14-18 years	696 (26.2%)	888 (33.5%)	1069 (40.3%)
**Co-occurring Critical Congenital Heart Defect (CCHD)**			
Yes	82 (23.3%)	117 (33.2%)	153 (43.5%)
No	1916 (24.4%)	2723 (34.7%)	3210 (40.9%)
**Maternal Age at Analysis** ^**#**^			
≤35	330 (22.4%)	433 (29.4%)	709 (48.2%)
>35	1668 (24.8%)	2407 (35.8%)	2654 (39.4%)
**Maternal Race/Ethnicity**			
White non-Hispanic	397 (16.5%)	1145 (47.6%)	863 (35.9%)
Black non-Hispanic	278 (37.7%)	320 (44.4%)	140 (19.0%)
Hispanic	1217 (26.1%)	1189 (25.4%)	2266 (48.5%)
Additional non-Hispanic groups	106 (27.6%)	184 (47.9%)	94 (24.5%)
**Maternal Education at Delivery**			
Greater than high school	758 (19.0%)	1611 (40.4%)	1617 (40.6%)
High school	541 (27.5%)	582 (29.6%)	845 (42.9%)
Less than high school	690 (31.3%)	634 (28.7%)	884 (40.0%)
**Maternal Residence at Delivery: Concentrated Poverty of >20%** ^**§**^			
Yes	601 (22.5%)	356 (17.8%)	1046 (52.2%)
No	1397 (22.5%)	2484 (40.1%)	2317 (37.4%)
**Maternal Residence at Delivery: Texas-Mexico Border/Non-Border**			
***14 Texas Counties***			
Border	0	0	1084 (100%)
Non-border	1998 (28.1%)	2840 (39.9%)	2279 (32.0%)
***32 Texas Counties***			
Border	*	0	1128 (99.8%)
Non-border	1997 (28.3%)	2839 (40.2%)	2234 (31.6%)
**Maternal Residence at Delivery: Urban/Rural**			
Urban	1991 (26.7%)	2581 (34.6%)	2897 (38.8%)
Rural	7 (0.1%)	259 (35.4%)	465 (63.6%)

† The data reflects individuals who, based on available information, are presumed to be living. This assumption is made with the understanding that the registry may not capture all changes in status over time.

# Analysis was completed in October 2024.

^ Percentages (%) may not total 100% due to rounding or missing data. This table presents descriptive distributions of categorized travel burden. Statistical comparisons were conducted using continuous one-way driving time measure and are reported in Table 1.

* Counts less than 5 were suppressed.

§ By census tract, 5-year estimates, American Community Survey 2023.

**Table 4:** Travel burden categories by median one-way driving distance for living individuals with DS^†^ by select characteristics, Texas, 1999-2020.

Characteristics	Travel Burden Categories by One-Way Driving Distance (N, %) ^		
	Low (≤ 30 miles)	Moderate to High (> 30 miles and ≤ 90 miles)	Very High (>90 miles)
**Individual with DS Age at Analysis** ^**#**^			
4-8 years	1172 (41.3%)	363 (12.8%)	1304 (45.9%)
9-13 years	1170 (43.2%)	326 (12.0%)	1213 (44.8%)
14-18 years	1188 (44.8%)	287 (10.8%)	1178 (44.4%)
**Co-occurring Critical Congenital Heart Defect (CCHD)**			
Yes	140 (39.8%)	48 (13.6%)	164 (46.6%)
No	3390 (43.2%)	928 (11.8%)	3531 (45.0%)
**Maternal Age at Analysis** ^**#**^			
≤35	535 (36.4%)	168 (11.4%)	769 (52.2%)
>35	2995 (44.5%)	808 (12.0%)	2926 (43.5%)
**Maternal Race/Ethnicity**			
White non-Hispanic	869 (36.1%)	513 (21.3%)	1023 (42.5%)
Black non-Hispanic	504 (68.3%)	60 (8.1%)	174 (23.6%)
Hispanic	1908 (40.8%)	366 (7.8%)	2398 (51.3%)
Additional non-Hispanic groups	247 (64.3%)	37 (9.6%)	100 (26.0%)
**Maternal Education at Delivery**			
Greater than high school	1653 (41.5%)	554 (13.9%)	1779 (44.6%)
High school	832 (42.3%)	209 (10.6%)	927 (47.1%)
Less than high school	1029 (46.6%)	207 (9.4%)	972 (44.0%)
**Maternal Residence at Delivery: Concentrated Poverty of >20%** ^**§**^			
Yes	794 (39.6%)	106 (5.3%)	1103 (55.1%)
No	2736 (44.2%)	870 (14.0%)	2592 (41.8%)
**Maternal Residence at Delivery: Texas-Mexico Border/Non-Border**			
***14 Texas Counties***			
Border	0	0	1084 (100%)
Non-border	3530 (49.6%)	976 (13.7%)	2611 (36.7%)
***32 Texas Counties***			
Border	*	0	1129 (99.9%)
Non-border	4250 (60.1%)	243 (3.4%)	2577 (36.5%)
**Maternal Residence at Delivery: Urban/Rural**			
Urban	3521 (47.1%)	799 (10.7%)	3149 (42.2%)
Rural	9 (1.2%)	177 (24.2%)	545 (74.6%)

† The data reflects individuals who, based on available information, are presumed to be living. This assumption is made with the understanding that the registry may not capture all changes in status over time.

# Analysis was completed in October 2024.

^ Percentages (%) may not total 100% due to rounding or missing data. This table presents descriptive distributions of categorized travel burden. Statistical comparisons were conducted using continuous one-way driving distance measure and are reported in Table 1.

* Counts less than 5 were suppressed.

§ By census tract, 5-year estimates, American Community Survey 2023.

**Table 5:** Travel burden categories by median one-way driving time for individuals with DS with post-neonatal death^‡^ by select characteristics, Texas, 1999-2020.

Characteristics	Travel Burden Categories by One-Way Driving Time (N, %) ^		
	Low (≤ 30 minutes)	Moderate to High (> 30 minutes and ≤ 2 hours)	Very High (>2 hours)
**Individual with DS Age at Death**			
29 days-5 years	91 (22.1%)	120 (29.2%)	200 (48.7%)
6 years-12 years	6 (16.2%)	14 (37.8%)	17 (46.0%)
13 years-20 years	7 (24.1%)	6 (20.7%)	16 (55.2%)
**Co-occurring Critical Congenital Heart Defect (CCHD)**			
Yes	*	*	*
No	102 (21.8%)	136 (29.0%)	231 (49.3%)
**Maternal Age at Death of Child with DS**			
≤35	66 (24.2%)	71 (26.0%)	136 (49.8%)
>35	38 (18.6%)	69 (33.8%)	97 (47.6%)
**Maternal Race/Ethnicity**			
White non-Hispanic	24 (17.5%)	58 (40.2%)	55 (42.3%)
Black non-Hispanic	20 (35.7%)	23 (41.1%)	13 (23.2%)
Hispanic	54 (20.3%)	50 (18.8%)	162 (60.9%)
Additional non-Hispanic groups	6 (33.3%)	9 (50.0%)	*
**Maternal Education at Delivery**			
Greater than high school	41 (24.9%)	41 (24.9%)	83 (50.3%)
High school	30 (22.6%)	31 (23.3%)	72 (54.1%)
Less than high school	32 (18.1%)	67 (37.9%)	78 (44.1%)
**Maternal Residence at Delivery: Concentrated Poverty of >20%** ^**§**^			
Yes	44 (22.7%)	40 (20.6%)	110 (56.7%)
No	60 (21.3%)	100 (35.5%)	122 (43.3%)
**Maternal Residence at Delivery: Texas-Mexico Border/Non-Border**			
***14 Texas counties***			
Border	0	0	77 (100.0%)
Non-border	104 (26.0%)	140 (35.0%)	156 (39.0%)
***32 Texas counties***			
Border	0	0	79 (100%)
Non-border	95 (23.9%)	115 (28.9%)	188 (47.2%)
**Maternal Residence at Delivery: Urban/Rural**			
Urban	0	15 (25.0%)	45 (75.0%)
Rural	104 (24.9%)	125 (30.0%)	188 (45.1%)

‡ The data reflects individuals who, based on available information, are deceased. This assumption is made with the understanding that the registry may not capture all changes in status over time.

^ Percentages (%) may not total 100% due to rounding or missing data. This table presents descriptive distributions of categorized travel burden. Statistical comparisons were conducted using continuous one-way driving time measure and are reported in Table 2.

* Counts less than 5 were suppressed.

§ By census tract, 5-year estimates corresponding to the year of death.

**Table 6:** Travel burden categories by median one-way driving distance for individuals with DS with post-neonatal death^‡^ by select characteristics, Texas, 1999-2020.

Characteristics	Travel Burden Categories by One-Way Driving Distance (N, %) ^		
	Low (≤ 30 miles)	Moderate to High (> 30 miles and ≤ 90 miles)	Very High (>90 miles)
**Individual with DS Age at Death**			
29 days-5 years	137 (33.3%)	59 (14.4%)	215 (52.3%)
6 years-12 years	15 (40.5%)	4 (10.8%)	18 (48.7%)
13 years-20 years	7 (35.7%)	2 (10.0%)	11 (55.0%)
**Co-occurring Critical Congenital Heart Defect (CCHD)**			
Yes	*	*	*
No	158 (33.7%)	63 (13.4%)	248 (52.9%)
**Maternal Age at Death of Child with DS**			
≤35	90 (33.0%)	35 (12.8%)	148 (54.2%)
>35	71 (34.8%)	30 (14.7%)	103 (50.5%)
**Maternal Race/Ethnicity**			
White non-Hispanic	38 (27.7%)	34 (24.8%)	65 (23.6%)
Black non-Hispanic	35 (62.5%)	7 (12.5%)	13 (23.6%)
Hispanic	75 (28.2%)	22 (8.3%)	169 (63.5%)
Additional non-Hispanic groups	13 (72.2%)	2 (11.1%)	3 (16.7%)
**Maternal Education at Delivery**			
Greater than high school	58 (35.2%)	18 (10.9%)	89 (53.9%)
High school	42 (37.6%)	14 (10.5%)	77 (57.9%)
Less than high school	60 (33.9%)	32 (18.1%)	85 (48.0%)
**Maternal Residence at Delivery: Concentrated Poverty of >20%** ^**§**^			
Yes	58 (29.9%)	20 (10.3%)	116 (59.8%)
No	103 (36.5%)	45 (16.0%)	134 (47.5%)
**Maternal Residence at Delivery: Texas-Mexico Border/Non-Border**			
***14 Texas Counties***			
Border	0	0	77(100%)
Non-border	161 (40.2%)	65 (16.3%)	174 (43.5%)
***32 Texas Counties***			
Border	0	0	79 (100%)
Non-border	100 (25.1%)	110 (27.6%)	188 (47.2%)
**Maternal Residence at Delivery: Urban/Rural**			
Urban	161 (38.6%)	54 (13.0%)	202 (48.4%)
Rural	0	11 (18.3%)	49 (81.7%)

‡ The data reflects individuals who, based on available information, are deceased. This assumption is made with the understanding that the registry may not capture all changes in status over time.

^ Percentages (%) may not total 100% due to rounding or missing data. This table presents descriptive distributions of categorized travel burden. Statistical comparisons were conducted using continuous one-way driving distance measure and are reported in Table 2.

* Counts less than 5 were suppressed.

§ By census tract, 5-year estimates corresponding to the year of death.

## Results

### Study population

Between 1999 and 2020, among 8,444,795 live births, there were a total of 11,694 individuals with DS identified in Texas. Of these, 8,201 individuals aged 18 years or younger were presumed living at the time of data linkage (October 2024). A separate cohort of 477 individuals with a documented post-neonatal death was also analyzed. The majority were born to Hispanic mothers (57%), followed by White NH (29%) and Black NH (9%) mothers. Most individuals were born to mothers residing in urban areas (91% among living, 87% among deceased). Greater than 40% of individuals with DS with post-neonatal death were born to mothers residing in high poverty (>20%) census tracts compared to 24% among living individuals. The demographic profiles of living and deceased cohorts were otherwise similar (Tables 1 and 2).

### Travel burden to pediatric DS specialty clinics for the living cohort

Seven pediatric DS specialty clinics that met the criteria (Fig 1) and were included in the analysis. Nearly half (45%) of living individuals with DS were required to travel more than 90 miles one way to reach the nearest specialty clinic, while 41% faced driving times exceeding two hours. (Tables 3 and 4; Figs 2 and 3).

**Fig 2:**
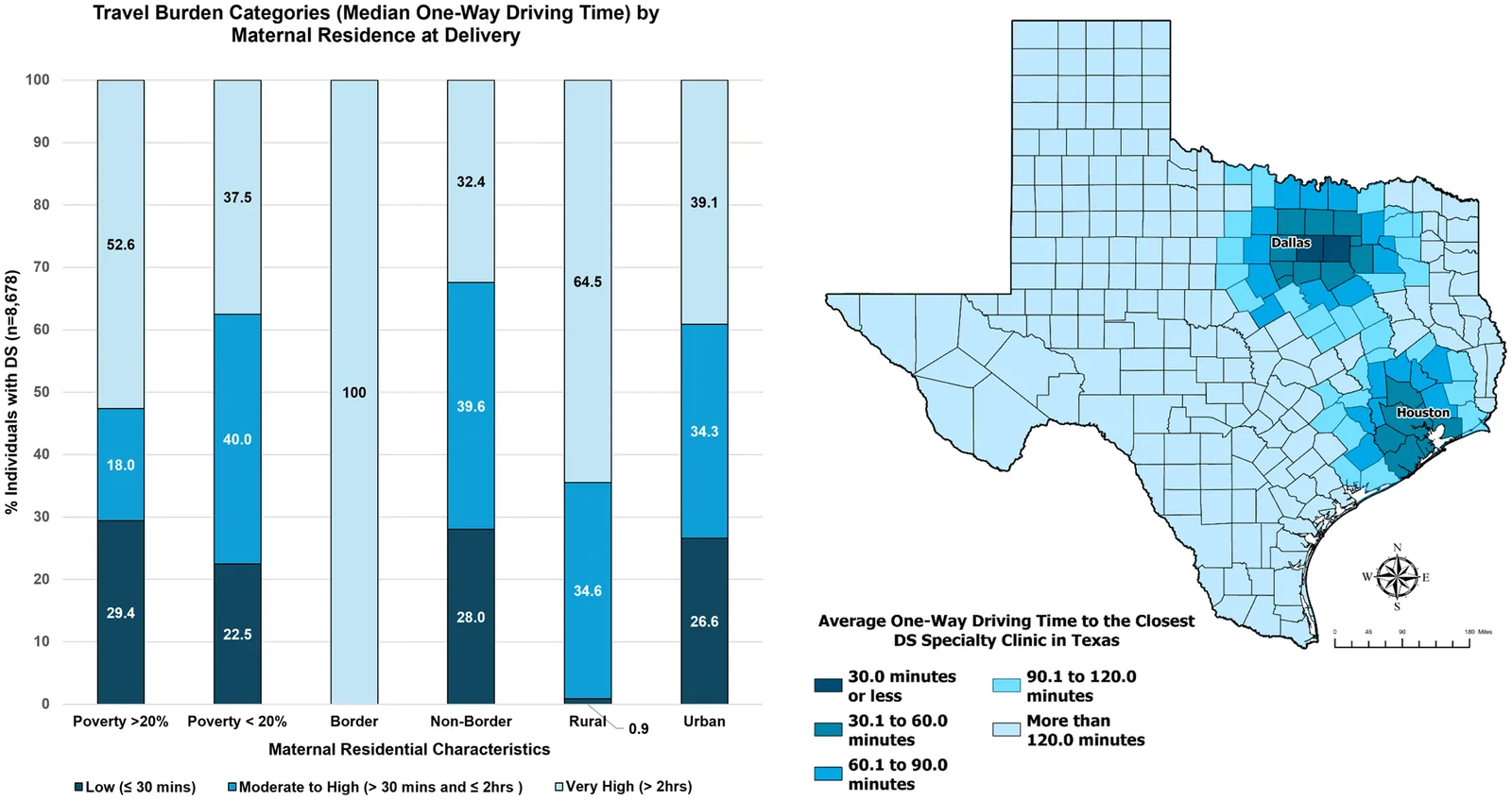
Average and median one-way driving time in minutes# to the closest DS specialty clinic in Texas for individuals born with DS* from 1999-2020 at county level. #One-way driving times to the closest DS specialty clinic were estimated using the ArcGIS Pro network analysis tool that considers all the feasible driving routes and roadway system including posted speed limits, intersection delays, one-way restrictions, and allowable turns. Base map derived from United States Census Bureau TIGER/Line shapefiles. *Includes both individuals with DS without any indication of death (n=8201) and individuals with DS with a documented indication of death (n = 477).

**Fig 3:**
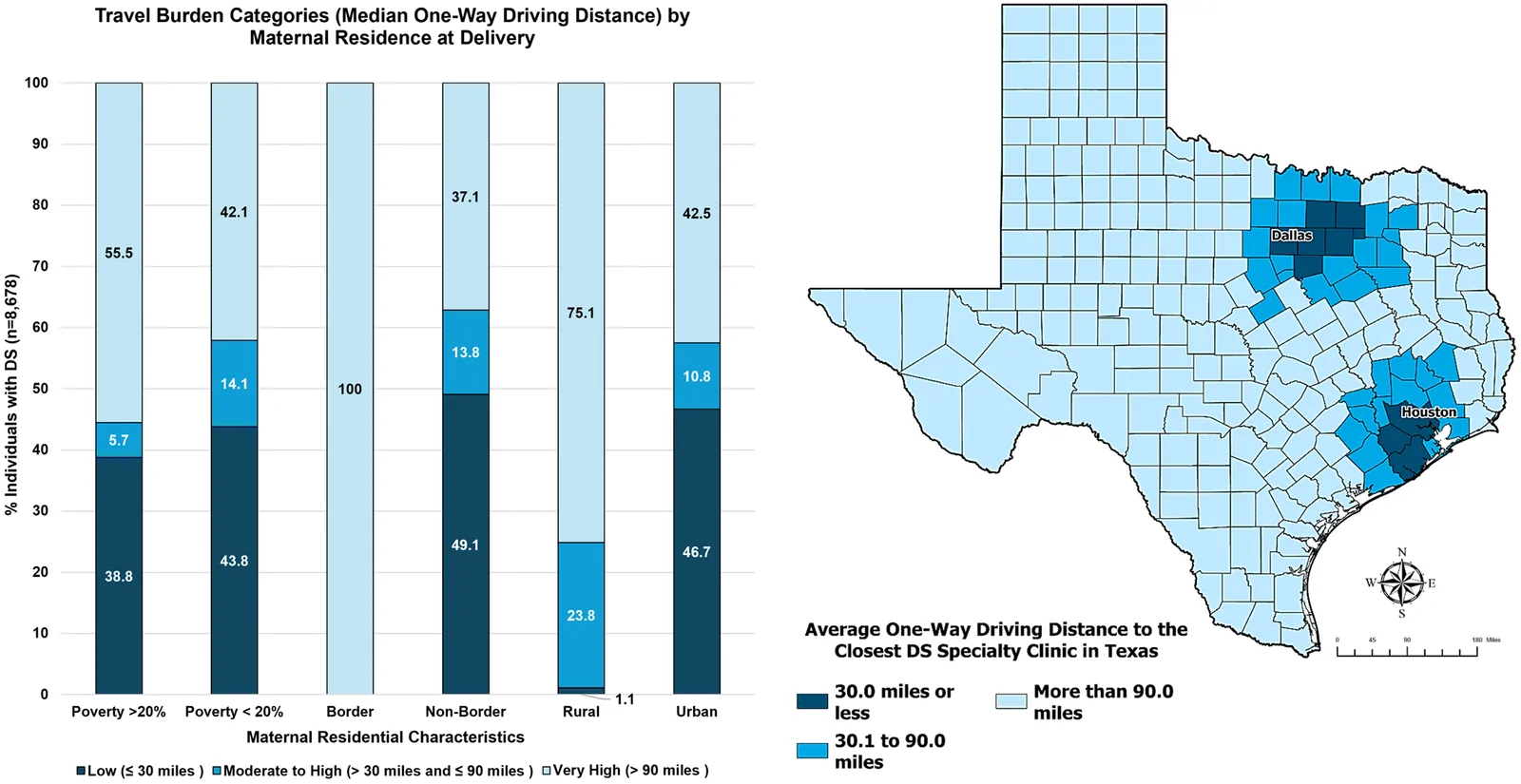
Average and median one-way driving distance in miles# to the closest DS specialty clinic in Texas for individuals born with DS* from 1999-2020 at county level. # One-way driving distances to the closest DS specialty clinic were estimated using the ArcGIS Pro network analysis tool that considers all the feasible driving routes and roadway system including posted speed limits, intersection delays, one-way restrictions, and allowable turns instead of straight-line distance. Base map derived from United States Census Bureau TIGER/Line shapefiles. *Includes both individuals with DS without any indication of death (n=8201) and individuals with DS with a documented indication of death (n = 477).

Substantial differences in access were observed across sociodemographic groups (Tables 1, 3 and 4). Median driving distance and time were significantly greater for individuals with DS born to Hispanic mothers (102.2 miles; 109.6 minutes) compared to White NH mothers (52.4; 67.3 minutes) and Black NH (19.6 miles; 34.6 minutes) mothers (p < 0.0001; Table 1). Nearly half of the individuals born to Hispanic mothers faced very high travel burden, with driving times exceeding two hours (Table 3). Among living individuals, maternal age at the time of analysis showed significant associations with travel burden measures (p < 0.0001; Table 1). Younger mothers faced median driving distances of 103.8 miles and times of 110.1 minutes (Table 1). Nearly half of younger mothers faced driving times exceeding two hours, compared to 39% of mothers aged 35 years and older (Table 3).

As seen in Figs 2 and 3, maternal urban residence was also strongly associated with lower travel burden (32.9 miles; 50.4 minutes) compared to rural residence (143.9 miles; 147.7 minutes) among the living cohort (p < 0.0001; Table 1). Similarly, individuals born to mothers residing in border counties faced very high travel burden with a median one-way driving time exceeding 400 minutes compared to less than 60 minutes for non-border residents (p < 0.0001; Table 1). Individuals with DS in high-poverty census tracts had significantly higher travel burden with median distances of 143.9 miles vs 37.2 miles, and time 138.1 minutes vs 54.8 minutes (p < 0.0001; Table 1) and 52% needed to travel more than two hours (Table 3; Fig 2). One-way median driving time and distance did not significantly differ by individual characteristics such as child’s age group or by the presence of a co-occurring CCHD (p > 0.05; Table 1).

### Travel burden to pediatric DS specialty clinics for the deceased cohort

Analysis of the deceased cohort revealed similar trends but with consistently higher travel burdens and more pronounced sociodemographic differences (Tables 2, 5 and 6). Nearly 50% of this group experienced one-way driving times greater than two hours. The sociodemographic differences were more pronounced in this cohort. The burden was particularly high for individuals born to Hispanic mothers, who faced a median driving time of 188.7 minutes, with 61% traveling over two hours (p < 0.0001; Tables 2 and 5). As seen in Figs 2 and 3, geographic divides appeared to widen further based on descriptive comparison; for instance, the median travel for rural residents in the deceased cohort (244.8 miles; 240.4 minutes) was markedly higher than for rural residents in the living cohort (143.9 miles; 147.7 minutes), and the urban-rural difference within the deceased cohort was also significant (p < 0.0001; Table 5). Residence in a high-poverty tract was also associated with a greater travel burden, with a median distance of 160.5 miles (p = 0.0030; Table 2) and median time of 165.1 minutes (p = 0.0019; Table 2), with 57% of this group traveling more than two hours (Table 5). While not statistically significant, individuals who died between ages 13–20 faced the highest travel burden in this cohort (Table 2). As with the living cohort, the presence of a co-occurring CCHD was not associated with travel burden.

## Discussion

In this population-based analysis of over 8,600 individuals with DS in Texas, United States, we identified substantial geographic and sociodemographic differences in geographic proximity to pediatric DS specialty clinics in Texas. Nearly half of the individuals in the living cohort would need to travel more than two hours one way to reach the nearest in-state specialty clinic. For children with DS, who often require ongoing monitoring and coordination across multiple services, travel times of this magnitude may represent a meaningful barrier to recommended care [5,6,16,57]. Travel burdens were highest among individuals born to Hispanic mothers and mothers residing in rural counties, the Texas-Mexico border, and high poverty census tracts. Even in urban areas, where most clinics are concentrated, a substantial number of individuals experienced moderate-to-high travel burden. Taken together, these findings suggest that the geographic distribution of pediatric DS specialty clinics in Texas does not align evenly with the distribution of the population in need. Very few studies have quantified access to DS specialty clinics at a population level [5,57], and, to our knowledge, none have done so using geospatial methods utilizing an active population-based surveillance registry. Our findings are broadly consistent with prior work showing that geographic access to DS specialty care is uneven in the United States [5,57]. However, this study extends literature by characterizing geographic proximity to these clinics within Texas, a particularly important setting because of its large geographic footprint, limited number of clinics, and substantial ethnic, linguistic, and socio-economic diversity [49,50]. Although some travel burden might be expected in a state this large, our findings indicate that the burden is not distributed equally across populations.

The greatest travel burden was observed among individuals born to mothers residing in rural counties and Texas-Mexico border counties (Figs 2 and 3). These differences arise largely because, as noted, all identified pediatric DS specialty clinics are clustered in major metropolitan areas, particularly Houston and the Dallas/Fort Worth region, leaving many families in rural and border counties without nearby DS specialty clinic services. This interpretation is consistent with broader literature showing that specialty and pediatric subspecialty services are often concentrated in urban centers, creating access barriers for rural populations and for children with special healthcare needs [6,40,42–45]. For instance, in 2013, Delmelle et al. found that children with birth defects in rural areas in the state of Florida frequently traveled much farther for specialty care, and that healthcare utilization patterns reflect these distances as urban families access more specialty services because they are closer to providers [6]. In that context, our findings suggest that centralized specialty care models can be associated with substantial burdens for families living far from metropolitan hubs.

Our findings also align with literature showing that pediatric subspecialty access is shaped by both geographic maldistribution and the organization of specialty-care networks. Prior studies have documented substantial regional variation in pediatric subspecialist supply, narrower pediatric than adult specialty networks, and persistent access barriers among children with special health care needs despite formal network adequacy standards [42,43,45–47]. The travel burdens observed in Texas likely reflect one component of a broader access landscape in which geographic proximity, provider availability, insurance networks, and referral patterns jointly influence whether children access and receive specialized care.

We also observed higher travel burden among individuals born to Hispanic mothers and mothers living in high-poverty census tracts. These findings likely reflect the intersection of geography and social factors rather than geography alone. Prior studies have documented racial and ethnic differences in time spent obtaining medical care and broader differences in healthcare access across rural and urban settings in the United States [43,58,59]. Other work has shown that socioeconomic conditions are associated with poorer access to care and lower healthcare utilization among children with special health care needs and other pediatric populations [60–62]. In Texas, South Texas has a large Hispanic population [63] and lacks DS specialty clinics, which likely explains the greater travel burden observed among individuals born to Hispanic mothers in this study. Among the living cohort, individuals born to younger mothers also experienced greater travel burden. This association may reflect broader social and geographic patterning rather than maternal age itself. Prior literature suggests that younger maternal age may be associated with lower socioeconomic status and with residence in areas that are farther from specialized services [64,65]. In addition, national data show that mothers in rural and nonmetropolitan counties tend to be younger at first birth than mothers in large metropolitan counties [66,67].

In secondary analyses, individuals with DS with a documented death generally resided farther from DS specialty clinics than those in the living cohort. These findings should be interpreted cautiously, as this study was not designed to evaluate the effect of geographic proximity of clinics on mortality and no causal conclusions can be drawn. Nonetheless, prior literature suggests that transportation barriers and greater distance to specialized care can be associated with poorer health outcomes, reduced healthcare utilization, and lower quality of life [58,68,69]. In the DS context, prior work has also documented gaps in awareness of and adherence to recommended care guidelines, particularly where structural and socioeconomic barriers exist [35,38]. Together, these findings support the need for future work examining whether geographic differences in access to care are associated with differences in service utilization and long-term health outcomes.

The study had several key strengths including the use of robust, population-based data from the TBDR, which employs comprehensive, active surveillance methodologies, ensuring accurate and representative findings. Advanced geospatial analytic techniques were employed to precisely estimate one-way driving time and distance, enhancing the reliability of the results. Specifically, the application of road network analysis instead of Euclidean or straight-line distances accounts for actual driving routes, speed limits, and one-way roads, thereby yielding a more accurate measure of real-world accessibility to DS specialty clinics. Additionally, the study’s comprehensive inclusion criteria, encompassing both living and deceased cohorts, provided detailed insights into geographic proximity dynamics.

Limitations of this study include a lack of individual-level data on actual care utilization or parental preferences, which may differ from geographic access alone. Our analysis estimates geographic proximity to identified pediatric DS specialty clinics in Texas rather than realized healthcare utilization, patient preference, or quality of care. Individuals with DS may receive guideline recommended care through primary care physicians, individual subspecialists, or in other multidisciplinary settings not meeting our definition of a DS specialty clinic. Thus, greater distance from a designated clinic should not be interpreted as evidence that appropriate care was unavailable or that individuals necessarily traveled to that clinic. Because this study focused specifically on DS specialty clinics, access to these other models of care was not evaluated. Claims-based or other longitudinal utilization data could complement these findings by identifying where individuals with DS receive care and whether proximity to a DS specialty clinic influences utilization, continuity, or outcomes. In addition, because residential data after delivery were unavailable, we used residence at delivery as a proxy for current residence. Maternal residential address at delivery represents a single point in time and may not reflect the child’s residence throughout childhood. Families may relocate after the birth of a child with DS, potentially in response to employment, family support, healthcare needs, or proximity to specialized services. Therefore, our estimates characterize geographic proximity based on residence at delivery and should not be interpreted as longitudinal measures of travel burden. Restricting the primary analysis to individuals aged 18 years or younger reduces concerns associated with using birth address for adult populations, it does not eliminate potential misclassification resulting from childhood residential mobility. Although research suggests that approximately 60% of young adults continue to live within a ten-mile radius of their birth residence [70] and in Texas population, maternal residential movement is generally within short distances [71]. Furthermore, this analysis did not assess other factors that affect accessibility like clinic capacity, clinic hours, insurance acceptance, wait time and referral pathways. Accordingly, geographic proximity should be interpreted as one component of access rather than a direct measure of realized access to care. Although these clinics were identified as DS-focused programs within larger pediatric hospital systems with access to relevant subspecialty referral networks, we did not assess whether additional travel beyond the nearest identified DS specialty clinic was required to obtain other needed services. Additionally, this analysis characterized geographic proximity to the pediatric DS specialty clinics located within Texas and did not include clinics in neighboring states or Mexico. For individuals residing near state or international boundaries, the closest DS specialty clinic may be located outside Texas. Our estimates may overstate the minimum travel required to reach any DS specialty clinic, particularly among border populations. Based on our manual verification of specialized networks in adjacent states, the closest out-of-state multidisciplinary pediatric DS specialty clinic at the time of this analysis was located approximately 140 miles away in Little Rock, Arkansas. Given this distance, it is less likely that omitting out-of-state centers significantly altered the primary geographic proximity metrics for the vast majority of our study population. Access to geographically proximate out-of-state or international care may also be influenced by barriers related to insurance networks and other system factors [46,47]. Public insurance programs like Texas Medicaid (including STAR Kids) and the Children’s Health Insurance Program (CHIP) may not cover routine, non-emergency specialized care outside of Texas boundaries without rare and highly restrictive single-case exceptions [72]. Similarly, private commercial payers frequently utilize localized or state-bounded provider networks. Future analyses incorporating clinics outside Texas and observed healthcare utilization could provide a more complete assessment of the care options available to these populations.

Another limitation is that our search methodology may not have captured pediatricians with expertise in DS care who practice in a solo or small group settings that lack a formal website or online listing. We also did not have data on potential mitigating factors such as transportation assistance programs or family resources. Telehealth clinics were not included in this study, as our primary goal was to assess the physical accessibility of DS specialty clinics. Many evaluations, screenings and therapies recommended by the AAP health supervision guidelines often necessitate in-person clinic visits for accurate diagnosis and effective intervention [34]. Finally, although advanced geospatial approaches were used, travel-time calculations cannot fully capture temporal variations such as rush-hour traffic or seasonal weather impacts on road conditions.

Despite these constraints, our results have population-level implications for healthcare administration and policy planning. We do not suggest that a dedicated DS specialty clinic should be established in every rural county or small town but identifying underserved areas may help guide the strategic deployment of alternative care models. For instance, our findings suggest that the regions experiencing the highest travel burdens, particularly along the Texas-Mexico border, would benefit from transport and lodging support and from expanded outreach through satellite or pop-up clinics in underserved areas. Additionally, strengthening the role of the patient’s medical home by equipping local providers with the training to co-manage care could help reduce travel burden for individuals who live far from centralized specialty hubs. While our analysis focused on Texas, these geographic differences in access likely reflect the reality of centralized healthcare models in other states and countries, where specialized care is concentrated in metropolitan areas [6,43,57,73,74]. Finally, although this study focused on DS specialty clinics, it would not be surprising if similar trends existed for other congenital conditions requiring multidisciplinary care. Further research is needed to explore life course residential history, evaluate actual clinic utilization, and assess access to other types of specialty clinics to better understand and address these geographic differences in access.

## Conclusion

We found that one-way driving distance and driving time to the nearest pediatric DS specialty clinic were greater for individuals born to Hispanic and younger mothers, and for those residing in rural areas, Texas-Mexico border counties, and concentrated-poverty regions. These findings suggest that travel burden associated with DS specialty care in Texas is not experienced uniformly across populations and may help inform future service planning.

## References

[1] Chung J, Donelan K, Macklin EA, Schwartz A, Elsharkawi I, Torres A. A randomized controlled trial of an online health tool about Down syndrome. Genet Med. 2021;23(1):163–73. doi: 10.1038/s41436-020-00952-7 32879436

[2] Skotko BG, Davidson EJ, Weintraub GS. Contributions of a specialty clinic for children and adolescents with Down syndrome. Am J Med Genet A. 2013;161A(3):430–7. doi: 10.1002/ajmg.a.35795 23401090

[3] Managing CF. Cystic Fibrosis Foundation. https://www.cff.org/managing-cf

[4] Mogayzel PJ Jr, Dunitz J, Marrow LC, Hazle LA. Improving chronic care delivery and outcomes: the impact of the cystic fibrosis Care Center Network. BMJ Qual Saf. 2014;23 Suppl 1:i3-8. doi: 10.1136/bmjqs-2013-002363 24608548

[5] Joslyn N, Berger H, Skotko BG. Geospatial Analyses of Accessibility to Down Syndrome Specialty Care. J Pediatr. 2020;218:146-150.e1. doi: 10.1016/j.jpeds.2019.10.058 31813535

[6] Delmelle EM, Cassell CH, Dony C, Radcliff E, Tanner JP, Siffel C, et al. Modeling travel impedance to medical care for children with birth defects using Geographic Information Systems. Birth Defects Res A Clin Mol Teratol. 2013;97(10):673–84. doi: 10.1002/bdra.23168 23996978 PMC4507419

[7] Lovett A, Haynes R, Sünnenberg G, Gale S. Car travel time and accessibility by bus to general practitioner services: a study using patient registers and GIS. Soc Sci Med. 2002;55(1):97–111. doi: 10.1016/s0277-9536(01)00212-x 12137192

[8] Bull MJ. Down Syndrome. N Engl J Med. 2020;382(24):2344–52. doi: 10.1056/NEJMra1706537 32521135

[9] Mai CT, Isenburg JL, Canfield MA, Meyer RE, Correa A, Alverson CJ, et al. National population-based estimates for major birth defects, 2010-2014. Birth Defects Research. 2019;111(18):1420–35. doi: 10.1002/bdr2.1589 PMC7203968 31580536

[10] Shumate C, Allred R, Dixon A, Betancourt D, Yantz C, Howell R, et al. Trends in the Prevalence of Down Syndrome (Trisomy 21) in Texas by Maternal Race/Ethnicity and Maternal Age Groups, 1999-2020. Am J Med Genet A. 2025;197(9):e64109. doi: 10.1002/ajmg.a.64109 PMC12339200 40320857

[11] Glasson EJ, Sullivan SG, Hussain R, Petterson BA, Montgomery PD, Bittles AH. The changing survival profile of people with Down’s syndrome: implications for genetic counselling. Clin Genet. 2002;62(5):390–3. doi: 10.1034/j.1399-0004.2002.620506.x 12431254

[12] Bittles AH, Glasson EJ. Clinical, social, and ethical implications of changing life expectancy in Down syndrome. Dev Med Child Neurol. 2004;46(4):282–6. doi: 10.1017/s0012162204000441 15077706

[13] Freeman SB, Bean LH, Allen EG, Tinker SW, Locke AE, Druschel C, et al. Ethnicity, sex, and the incidence of congenital heart defects: a report from the National Down Syndrome Project. Genet Med. 2008;10(3):173–80. doi: 10.1097/GIM.0b013e3181634867 18344706

[14] Dimopoulos K, Kempny A. Patients with Down syndrome and congenital heart disease: survival is improving, but challenges remain. Heart. 2016;102(19):1515–7. doi: 10.1136/heartjnl-2016-309835 27354276

[15] Bergström S, Carr H, Petersson G, Stephansson O, Bonamy A-KE, Dahlström A, et al. Trends in Congenital Heart Defects in Infants With Down Syndrome. Pediatrics. 2016;138(1):e20160123. doi: 10.1542/peds.2016-0123 27252035

[16] Skelton B, Knafl K, Van Riper M, Fleming L, Swallow V. Care Coordination Needs of Families of Children with Down Syndrome: A Scoping Review to Inform Development of mHealth Applications for Families. Children (Basel). 2021;8(7):558. doi: 10.3390/children8070558 34209506 PMC8304112

[17] Carfì A, Antocicco M, Brandi V, Cipriani C, Fiore F, Mascia D, et al. Characteristics of adults with down syndrome: prevalence of age-related conditions. Front Med (Lausanne). 2014;1:51. doi: 10.3389/fmed.2014.00051 25593924 PMC4292207

[18] Startin CM, D’Souza H, Ball G, Hamburg S, Hithersay R, Hughes KMO. Health comorbidities and cognitive abilities across the lifespan in Down syndrome. J Neurodev Disord. 2020;12(1):4. doi: 10.1186/s11689-019-9306-9 PMC6979347 31973697

[19] Altuna M, Giménez S, Fortea J. Epilepsy in Down Syndrome: A Highly Prevalent Comorbidity. J Clin Med. 2021;10(13):2776. doi: 10.3390/jcm10132776 PMC8268854 34202784

[20] Lott IT, Head E. Dementia in Down syndrome: unique insights for Alzheimer disease research. Nat Rev Neurol. 2019 Mar;15(3):135–47. doi: 10.1038/s41582-018-0132-6 PMC8061428 30733618

[21] Iulita MF, Garzón Chavez D, Klitgaard Christensen M, Valle Tamayo N, Plana-Ripoll O, Rasmussen SA, et al. Association of Alzheimer Disease With Life Expectancy in People With Down Syndrome. JAMA Netw Open. 2022;5(5):e2212910. doi: 10.1001/jamanetworkopen.2022.12910 35604690 PMC9127560

[22] Smythe T, Moyo-Chilufya M, Wilbur J, Greenland K, Engelbrecht A, Kumar M, et al. Advancing health-care access to optimise health and quality of life for children and young people with Down syndrome worldwide. Lancet Child Adolesc Health. 2026;10(9):668–78. doi: 10.1016/S2352-4642(26)00122-7 42190671

[23] Global Down Syndrome Foundation. 2026. https://www.globaldownsyndrome.org/research-medical-care/medical-care-providers/

[24] Jensen KM, Taylor LC, Davis MM. Primary care for adults with Down syndrome: adherence to preventive healthcare recommendations. J Intellect Disabil Res. 2013;57(5):409–21. doi: 10.1111/j.1365-2788.2012.01545.x 22463763

[25] Lee N-C, Chien Y-H, Hwu W-L. Integrated care for Down syndrome. Congenit Anom (Kyoto). 2016;56(3):104–6. doi: 10.1111/cga.12159 26866291

[26] Wexler ID, Abu-Libdeh A, Kastiel Y, Nimrodi A, Kerem E, Tenenbaum A. Optimizing health care for individuals with Down syndrome in Israel. Isr Med Assoc J. 2009;11(11):655–9. 20108550

[27] Peters VJT. Multidisciplinary Care for Children with Down syndrome in the Netherlands: A Modular Perspective. MRAJ. 2023;11(3). doi: 10.18103/mra.v11i3.3531

[28] van Gameren-Oosterom HBM, Weijerman ME, van Wieringen H, de Winter JP, van Wermeskerken AM. Clinical practice - latest insights in optimizing the care of children with Down syndrome. Eur J Pediatr. 2023;182(5):2027–39. doi: 10.1007/s00431-023-04890-9 36897403

[29] Schoonraad L, Slogrove A, Engelbrecht A, Urban MF. A 5-Year Retrospective Review of the Health Supervision Received by Children with Down Syndrome at a South African Regional Hospital. J Trop Pediatr. 2020;66(4):441–7. doi: 10.1093/tropej/fmz087 31943102

[30] Tenenbaum A, Kastiel Y, Meiner Z, Kerem E. Multidisciplinary care of persons with Down syndrome in Jerusalem. International Journal on Disability and Human Development. 2008;7(3). doi: 10.1515/ijdhd.2008.7.3.355

[31] Heerensperger D. Provision of coordinated care for individuals with Down syndrome: the Calgary perspective. Downs Syndr Res Pract. 2006;10(1):4–9. doi: 10.3104/case-studies.300 16869368

[32] Corder JP, Al Ahbabi FJS, Al Dhaheri HS, Chedid F. Demographics and co-occurring conditions in a clinic-based cohort with Down syndrome in the United Arab Emirates. Am J Med Genet A. 2017;173(9):2395–407. doi: 10.1002/ajmg.a.38338 28686324

[33] Sothirasan K, Anand AJ, Chua MPW, Khoo PC, Chien Chua M. Clinical Guideline for Management of Down Syndrome in Singapore. Proceedings of Singapore Healthcare. 2022;31. doi: 10.1177/20101058221104582

[34] Bull MJ, Trotter T, Santoro SL, Christensen C, Grout RW, COUNCIL ON GENETICS, et al. Health Supervision for Children and Adolescents with Down Syndrome. Pediatrics. 2022;149(5):e2022057010. doi: 10.1542/peds.2022-057010 35490285

[35] VanZant JS, Vellody K. Financial impact of a specialized Down syndrome clinic: Implications and support for institutional support of specialty care clinics. Am J Med Genet A. 2023;191(3):770–5. doi: 10.1002/ajmg.a.63072 36478040

[36] Hickey F, Wolter‐Warmerdam K, Winders P, Holland S, Kelminson K, Daniels D. Ten‐year impact of a Down syndrome pediatric clinic. Policy Practice Intel Disabi. 2023;20(4):371–9. doi: 10.1111/jppi.12471

[37] Eaves L, Ho H, Laird B, Dickson S. Raising a child with Down syndrome: Parents’ evaluations of health information and support. Downs Syndr Res Pract. 1996;4(2):65–9. doi: 10.3104/reports.64

[38] Santoro SL, Martin LJ, Pleatman SI, Hopkin RJ. Stakeholder Buy-In and Physician Education Improve Adherence to Guidelines for Down Syndrome. J Pediatr. 2016;171:262-8.e1-2. doi: 10.1016/j.jpeds.2015.12.026 26831529

[39] Kelly C, Hulme C, Farragher T, Clarke G. Are differences in travel time or distance to healthcare for adults in global north countries associated with an impact on health outcomes? A systematic review. BMJ Open. 2016;6(11):e013059. doi: 10.1136/bmjopen-2016-013059 27884848 PMC5178808

[40] Mseke EP, Jessup B, Barnett T. Impact of distance and/or travel time on healthcare service access in rural and remote areas: A scoping review. Journal of Transport & Health. 2024;37:101819. doi: 10.1016/j.jth.2024.101819

[41] Wolfe MK, McDonald NC, Holmes GM. Transportation Barriers to Health Care in the United States: Findings from the National Health Interview Survey, 1997-2017. Am J Public Health. 2020;110(6):815–22. doi: 10.2105/AJPH.2020.305579 PMC7204444 32298170

[42] Mayer ML. Are we there yet? Distance to care and relative supply among pediatric medical subspecialties. Pediatrics. 2006;118(6):2313–21. doi: 10.1542/peds.2006-1570 17142513

[43] Turner A, Ricketts T, Leslie LK. Comparison of Number and Geographic Distribution of Pediatric Subspecialists and Patient Proximity to Specialized Care in the US Between 2003 and 2019. JAMA Pediatr. 2020;174(9):852–60. doi: 10.1001/jamapediatrics.2020.1124 32421165 PMC7235911

[44] Cull WL, Burr WH, Somberg CA, Olson LM. Primary Care Pediatrician Assessment of Access Barriers and Local Supply of Pediatric Subspecialists. Acad Pediatr. 2026;26(6):103342. doi: 10.1016/j.acap.2026.103342 42288151

[45] Maleki S, Dede-Bamfo N, Ekren E, Mohammadalizadehkorde M, Villagran M. Mapping Access to Children’s Hospitals in Texas. Int J Environ Res Public Health. 2024;21(2):140. doi: 10.3390/ijerph21020140 38397631 PMC10888133

[46] Wong CA, Kan K, Cidav Z, Nathenson R, Polsky D. Pediatric and adult physician networks in Affordable Care Act marketplace plans. Pediatrics. 2017;139(4):e20163117. doi: 10.1542/peds.2016-3117 28250022

[47] Hu J-C, Cummings JR, Ji X, Wilk AS. State-Level Variation in Medicaid Managed Care Enrollment and Specialty Care for Publicly Insured Children. JAMA Netw Open. 2023;6(10):e2336415. doi: 10.1001/jamanetworkopen.2023.36415 37796501 PMC10556966

[48] Cholera R, Anderson DM, Chung R, Genova J, Shrader P, Bleser WK, et al. Analysis of North Carolina Medicaid Claims Data to Simulate a Pediatric Accountable Care Organization. JAMA Netw Open. 2023;6(8):e2327264. doi: 10.1001/jamanetworkopen.2023.27264 37540515 PMC10403786

[49] Texas Department of State Health Services. The Texas Portion of the U.S.-Mexico Border. https://www.dshs.texas.gov/regional-local-health-operations/texas-border-health/tx-mx-border

[50] U.S. Census Bureau. QuickFacts: Texas. https://www.census.gov/quickfacts/fact/table/TX/PST045225

[51] Miller E. Evaluation of the Texas Birth Defects Registry: an active surveillance system. Birth Defects Res A Clin Mol Teratol. 2006;76(11):787–92. doi: 10.1002/bdra.20331 17094140

[52] Faure E, Danjou AMN, Clavel-Chapelon F, Boutron-Ruault MC, Dossus L, Fervers B. Accuracy of two geocoding methods for geographic information system-based exposure assessment in epidemiological studies. Environ Health. 2017;16(1):15. doi: 10.1186/s12940-017-0217-5 PMC5324215 28235407

[53] Thompson LK, Langholz B, Goldberg DW, Wilson JP, Ritz B, Tayour C, et al. Area-Based Geocoding: An Approach to Exposure Assessment Incorporating Positional Uncertainty. Geohealth. 2021;5(12):e2021GH000430. doi: 10.1029/2021GH000430 34859166 PMC8612311

[54] Identify the closest facility—ArcGIS Pro | Documentation. https://pro.arcgis.com/en/pro-app/3.4/help/analysis/networks/closest-facility-tutorial.htm

[55] Rural-Urban Continuum Codes | Economic Research Service. https://www.ers.usda.gov/data-products/rural-urban-continuum-codes

[56] U.S. Census Bureau. American Community Survey, 5-Year Estimates, Table S1701: Poverty Status in the Past 12 Months. https://census.gov

[57] Jensen K, Llamas P, Howard J, Berger H, Skotko BG. Specialty Care Oases: Utilizing Geographic Information Systems to Evaluate Specialty Care Access for Individuals With Down Syndrome. Am J Med Genet A. 2025;197(5):e63977. doi: 10.1002/ajmg.a.63977 39739375

[58] Ray KN, Chari AV, Engberg J, Bertolet M, Mehrotra A. Disparities in Time Spent Seeking Medical Care in the United States. JAMA Intern Med. 2015;175(12):1983–6. doi: 10.1001/jamainternmed.2015.4468 26437386 PMC5055855

[59] Caldwell JT, Ford CL, Wallace SP, Wang MC, Takahashi LM. Intersection of Living in a Rural Versus Urban Area and Race/Ethnicity in Explaining Access to Health Care in the United States. Am J Public Health. 2016;106(8):1463–9. doi: 10.2105/AJPH.2016.303212 27310341 PMC4940644

[60] Ganguly AP, Alvarez KS, Mathew SR, Soni V, Vadlamani S, Balasubramanian BA, et al. Intersecting social determinants of health among patients with childcare needs: a cross-sectional analysis of social vulnerability. BMC Public Health. 2024;24(1):639. doi: 10.1186/s12889-024-18168-8 38424507 PMC10902938

[61] Judge AS, Downing KF, Nembhard WN, Oster ME, Farr SL. Racial and ethnic disparities in socio-economic status, access to care, and healthcare utilisation among children with heart conditions, National Survey of Children’s Health 2016-2019. Cardiol Young. 2023;33(12):2539–47. doi: 10.1017/S1047951122004097 36999847 PMC10680441

[62] Mayer ML, Skinner AC, Slifkin RT. Unmet need for routine and specialty care: data from the National Survey of Children with Special Health Care Needs. Pediatrics. 2004;113(2):e109-115. doi: 10.1542/peds.113.2.e109 14754979

[63] The South Texas Region. https://comptroller.texas.gov/economy/economic-data/regions/2018/south.php

[64] Golestani R, Farahani FK, Peters P. Exploring barriers to accessing health care services by young women in rural settings: a qualitative study in Australia, Canada, and Sweden. BMC Public Health. 2025;25(1):213. doi: 10.1186/s12889-025-21387-2 PMC11742782 39825291

[65] Rural households generally have lower housing expenses relative to their income compared to urban households. Economic Research Service. https://ers.usda.gov/data-products/charts-of-note/chart-detail?chartId=76781

[66] Ely DM, Hamilton BE. Trends in fertility and mother’s age at first birth among rural and metropolitan counties: United States, 2007-2017. 2018. 30475685

[67] Brown AD, Hamilton BE, Kissin DM, Martin JA. Trends in Mean Age of Mothers in the United States, 2016 to 2023. Natl Vital Stat Rep. 2025;74(9):1–6. doi: 10.15620/cdc/174598 PMC12278045 40570233

[68] Fixler DE, Nembhard WN, Xu P, Ethen MK, Canfield MA. Effect of acculturation and distance from cardiac center on congenital heart disease mortality. Pediatrics. 2012;129(6):1118–24. doi: 10.1542/peds.2011-3114 22566422

[69] Syed ST, Gerber BS, Sharp LK. Traveling towards disease: transportation barriers to health care access. J Community Health. 2013;38(5):976–93. doi: 10.1007/s10900-013-9681-1 23543372 PMC4265215

[70] Bureau UC. New data tool and research show where people move as young adults. Census.gov. https://www.census.gov/library/stories/2022/07/theres-no-place-like-home.html

[71] Lupo PJ, Symanski E, Chan W, Mitchell LE, Waller DK, Canfield MA, et al. Differences in exposure assignment between conception and delivery: the impact of maternal mobility. Paediatr Perinat Epidemiol. 2010;24(2):200–8. doi: 10.1111/j.1365-3016.2010.01096.x 20415777

[72] Texas Medicaid Provider Procedures Manual. 2026. https://www.tmhp.com/resources/provider-manuals/tmppm

[73] McEvoy CS, Ross-Li D, Norris EA, Ricca RL, Gow KW. From far and wide: Geographic distance to pediatric surgical care across Canada. J Pediatr Surg. 2020;55(5):908–12. doi: 10.1016/j.jpedsurg.2020.01.036 32063366

[74] Terliesner N, Lesniowski D, Krasnikova A. Geographical accessibility of pediatric inpatient, nephrology, and urology services in Europe. Front Pediatr. 2020;8:395. doi: 10.3389/fped.2020.00395 PMC7396594 32850526

